# Social Comparison Effects on Academic Self-Concepts—Which Peers Matter Most?

**DOI:** 10.1037/dev0001368

**Published:** 2022-04-25

**Authors:** Malte Jansen, Zsófia Boda, Georg Lorenz

**Affiliations:** 1 Institute for Educational Quality Improvement (IQB), Humboldt University Berlin; 2 Centre for International Student Assessment (ZIB), Munich, Germany; 3 Department of Sociology and Institute for Social and Economic Research (ISER), University of Essex

**Keywords:** academic self-concept, big fish little pond effect, social comparison, social network analysis, stochastic actor-oriented models

## Abstract

Social comparisons with peers are important sources of self-development during adolescence. Many previous studies showed that students’ academic self-concepts (ASC) form by contrasting one’s own achievement with the average of one’s class or school (the Big-Fish-Little-Pond Effect [BFLPE]). Based on social comparison theory, however, we would expect some peers to be more likely social comparison targets than other peers, for example, because they are more visible or students perceive them as similar to themselves. In this study, we used sociometric data to analyze which peers play the most important role for social comparison effects on ASC. We examined how the average achievement of friends, study partners, peers perceived as popular by the student, as well as same-gender and same-ethnic peers affect the general ASC and how these effects compare to the effect of the classroom’s average achievement. The study was based on a German longitudinal sample of 2,438 students (44% no recent immigrant background, 19% Turkish immigrant background, 10% Eastern European immigrant background, 27% other immigrant background) from 117 school classes that were followed from grade 9 to 10. Results from longitudinal social network analysis do not confirm substantial incremental effects of specific types of peers, while class average achievement showed a stable negative effect (confirming the BFLPE). In addition, we could provide evidence for social selection effects based on ASC. We conclude that classrooms provide a specific setting that imposes social comparisons with the “generalized peer” rather than with specific subgroups of peers.

Students with high academic self-concepts (ASCs)—that is, students who think they are doing well at school—also show higher achievement, effort, attainment, academic aspirations, and intrinsic motivation (Denissen et al., 2007; Guo et al., 2017; Huang, 2011; Marsh et al., 2018; Valentine et al., 2004; Wigfield & Eccles, 2000). ASC is therefore a central component of academic motivation and one of the most studied motivational constructs in developmental and educational psychology. The development of students’ ASCs is not only shaped by their own achievement, but also by the social environment (Becker & Neumann, 2016; Gore & Cross, 2014; Huguet et al., 2009). The Big-Fish-Little-Pond Effect (BFLPE) predicts a higher ASC for a student in a lower-achieving class or school, compared with a student with the same ability level in a higher achieving class or school. Evidence for the BFLPE exists cross-culturally, in different domains, and for different age groups (Fang et al., 2018).

The BFLPE is assumed to result from social comparisons (Huguet et al., 2009; Marsh, 1987). One key assumption of the BFLPE is that students compare themselves with a “generalized other” (i.e., the average achievement of their classmates) to estimate their ranking within a given group (Huguet et al., 2009). Following this idea, all peers are assumed to be equally important social comparison targets^1^ because they contribute equally to the group average. However, this assumption can be questioned on the basis of insights from social comparison research, which emphasizes that information may not be equally available about all school- or classmates (Mussweiler, 2003) and that not all peers are equally subjectively meaningful for students (Lomi et al., 2011). Some peers, such as friends, peers one frequently studies with, particularly visible and popular peers, and peers with the same gender and ethnicity, may be more important comparison targets than other peers. Despite first attempts (see Koivuhovi et al., 2020; Wouters et al., 2013), though, the relative importance of such (probably overlapping) subgroups of peers as social comparison targets has rarely been tested in BFLPE research.

In this study, we examined which peers play the most important role for social comparison effects on ASC. We used sociometric data (i.e., information on friendship ties, on study partners, and on popularity perceptions) which we analyzed using longitudinal social network analyses techniques (stochastic actor-oriented models [SAOMs]). These models have only recently been introduced to the field of development psychology (Dijkstra et al., 2013; Gremmen et al., 2017; Laninga-Wijnen et al., 2019; Rambaran et al., 2017) and have a strong potential for gaining a deeper understanding of social comparison processes that has not yet been exploited in ASC research. This method allows us to investigate whether different types of peers impose social influence (i.e., students being affected by the characteristics of their peers as a result of comparisons with their peers’ achievement). At the same time, the SAOM approach considers that specific peers (e.g., friends and study partners) are self-selected in the first place and controls for such social selection processes (e.g., students with similar achievement or ASC selecting each other as friends) when estimating peer effects.

## Theoretical Background

### Social Comparison Theory

In his seminal Social Comparison Theory (SCT), Festinger (1954) postulated some core processes governing social comparisons. Among others, SCT introduced the *similarity hypothesis*: people should choose social comparison targets that are relatively similar to them in terms of achievement level or attitudes because such comparisons are perceived to be more informative and meaningful than comparisons with different targets (Festinger, 1954).

Since the first formulation of Festinger’s SCT, research on the conditions, mechanisms, and effects of social comparisons has been a staple in social psychology and also received attention in developmental psychology (Butler, 1998; France-Kaatrude & Smith, 1985). One central distinction added to SCT is that between contrasting and assimilating social comparisons (Gerber et al., 2018; Mussweiler, 2003). In the case of *contrast effects*, the ability self-perception diverges from the perceived ability of the target. That is, if the comparison target has a higher (lower) ability, one’s ASC will become lower (higher). In the case of *assimilation*, the ability self-perception adapts toward the ability of the target. That is, if the comparison target has a higher (lower) ability, one’s ASC would also become higher (lower). Assimilation has also been assumed to result from “basking in reflected glory” (Dockx et al., 2019; Trautwein et al., 2006) and indicates identification with a certain group and its characteristics. That is, students who are part of high-achieving groups and engage in assimilation, would conclude that owing to being part of this group, they must be good achievers, too.

Mussweiler (2003) argued that whether contrast or assimilation takes place depends on an initial, rapid, and holistic judgment of similarity between oneself and the target. If the target is perceived to be similar (e.g., if the target is a friend who shares characteristics with oneself), the subject will engage in similarity testing and knowledge relating to similarity will become more easily accessible leading to assimilating comparisons. On the contrary, if a target is perceived to be dissimilar in the initial judgment, the subject will engage in dissimilarity testing and information on dissimilarity is more easily accessible leading to contrasting comparisons. A recent meta-analysis by Gerber et al. (2018) reviewed more than 60 years of research on SCT. They focused on (mostly experimental) studies which involved comparisons to specific single targets (rather than multiple targets or group averages). The analysis showed a general tendency toward contrasting rather than assimilating social comparisons. The authors concluded that “the common response to comparison is contrast” (p. 18). As we will discuss below, this preference for contrasting comparisons is very consistent with the BFLPE.

### Social Comparisons in the Classroom: The Big-Fish-Little-Pond Effect

Students who learn in high-achieving groups (i.e., schools or classrooms) report lower ASCs than students with similar individual achievement in low-achieving learning groups. This is the central postulation of the BFLPE (Marsh, 1987) and one of the most robust findings in educational psychology (Seaton et al., 2009). Typical studies on the BFLPE use a multilevel design and large-scale observational data of students. The effect is then operationalized as the incremental negative effect of school- or classroom average achievement on ASC beyond the positive effect of individual achievement.

The BFLPE has been interpreted as a result of contrasting social comparison processes (Marsh, 1987). Testing the mechanism underlying this assumption (e.g., by testing boundary conditions of the effect) played a relatively minor role in early BFLPE research. In their critical review, Dai and Rinn (2008) suggested to broaden the research scope. Consequently, an increasing number of studies on the BFLPE began to test specific assumptions from SCT or used innovative research designs to replicate the effect. For example, some studies examined students that experienced a change in their learning environment (e.g., moving between school tracks or courses) and thus, assumingly, their frame of reference. These studies supported that students moving into higher-achieving contexts experienced a drop in their ASC (Arens & Watermann, 2015; von Keyserlingk et al., 2019; Wouters et al., 2012).

*Local dominance effects*, initially described in social psychology as the tendency to “rely on the most local comparison information while deemphasizing more general, and typically more diagnostic, forms of comparison feedback” (Zell & Alicke, 2010, p. 369), also received recent attention in BFLPE research. Several studies showed that the more local frame of reference of the classroom produced a stronger negative BFLPE than the school frame of reference (Janssen et al., 2015; Liem et al., 2013; Marsh et al., 2014). This is plausible given that (a) students know more about the achievement of their classmates compared with other peers in their school and (b) some teachers grade “on a curve” within a class and thus teacher-assigned grades, which are an important source of ASC (Marsh et al., 2018), partly represent the class rank.

Probably the most direct tests of social comparison mechanisms in BFLPE research were conducted in the few studies that combined estimations of the BFLPE with items asking students explicitly about their social comparison targets and about their perceived ability rank in their class. The seminal study by Huguet et al. (2009) showed that the substantial negative BFLPE (i.e., the usage of peer average achievement as a frame of reference for contrasting comparisons) coexists with additional single comparisons to specific classmates, which produce small assimilation effects. However, other salient characteristics that might affect the selection of comparison targets, such as gender or ethnic status, were not examined. Furthermore, the study showed that the BFLPE can be explained by students’ perceived rank in class. This study provided the most conclusive evidence for the notion that the BFLPE is based on social comparisons, more specifically “ego-deflating comparisons with the class standard” (Huguet et al., 2009, p. 158). The mediating effect of perceived within-class rank between average achievement and ASC has also been found in other studies (Marsh et al., 2008, 2014; Thijs et al., 2010).

This evidence suggests that social comparisons with generalized others (i.e., the average classmate), which lead to stable contrast effects, can be differentiated from social comparisons with specific others and that both processes can have independent effects. However, few previous studies investigated who these specific others are—that is, whether there are particular subgroups within a class that students compare themselves with.

## Social Comparisons With Specific Peers as Social Comparison Targets

In the following, we will describe three interrelated aspects that might drive social comparisons with specific groups of classmates as well as preferences among students for “local” frames of reference.

### Availability of Information on the Target’s Performance

To be able to conduct comparisons, students first need to know about the achievement of their target and that knowledge needs to be available to them when making their comparisons (Mussweiler, 2003). Students will know more about the abilities of students they frequently spend time with, which makes comparisons more accurate. This is an argument for the importance of friends and particularly study partners with whom students frequently engage in academic content.

### Similarity

The notion that people select similar others as preferred social comparison targets (similarity hypothesis) was already introduced by Festinger (1954) and its importance highlighted by Mussweiler (2003). Similar others could be peers from students’ social ingroups along salient dimensions, such as gender or ethnicity (Huguet et al., 2001; Tajfel, 1974). In addition, social network studies demonstrated that friends are more likely than other peers to show similarities along various dimensions (*homophily*) (McPherson et al., 2001), such as academic achievement (Gremmen et al., 2017; Kretschmer et al., 2018; Lomi et al., 2011), aspirations (Lorenz et al., 2020), favorite subjects (Raabe et al., 2019), and engagement (Wang et al., 2018). Therefore, friends may serve as “routine standards” for social comparisons (Lubbers et al., 2009; Mussweiler & Rüter, 2003). Similarities have also been found among those who study together (Stadtfeld et al., 2019), likely because study partners are also more likely to become friends over time and vice versa (Palacios et al., 2019), making study partners another likely comparison target.

### Status and (Perceived) Popularity

Social status has high importance in the life of adolescents. Students of high status can exert social power over other students by serving as opinion leaders or role models (Coleman, 1961), often through a combination of prosocial and aggressive behaviors (Cillessen et al., 2011). Therefore, other students might strive to adapt their behavior to that of peers they perceive as popular (Helms et al., 2014; Laninga-Wijnen et al., 2018). In the case of social comparisons, assimilation effects could be a means to increase similarity between oneself and popular students—if popular students had high academic achievement, other students may increase their ASCs. However, this would only apply for students who themselves have a high popularity motive which is not the case for all students (Dawes & Xie, 2017; Jones & Cooke, 2021).

### Summary and State of Research

Overall, it is unclear whether comparisons with specific peers would result in contrast or assimilation effects. Zell and Alicke (2010, p. 376) formulated a possible preference for friends within the framework of the above-mentioned local dominance effect and suggested that contrast effects would be present: “Students who, by chance, associate with friends […] who perform terribly on standardized tests may have inflated ability perceptions.” Furthermore, as mentioned above, previous research (Gerber et al., 2018) indicates that contrast effects are much more frequent and stable than assimilation effects. However, a perception of similarity to the target should increase the likelihood of assimilation—thus if assimilation effects do occur, it should be when comparing oneself with friends (Mussweiler, 2003; Suls et al., 2002).

Very few studies have tested the role of friends for within-class social comparisons and students’ self-concepts empirically. In longitudinal studies, having friends with high grades seemed to have a small detrimental effect on self-evaluations of low-achieving students (Altermatt & Pomerantz, 2005; Guay et al., 1999). However, the studies did not control for class-average achievement.

The probably most comprehensive study of Wouters et al. (2013) tested two competing hypotheses: a local frame of reference should result in “friendship dominance,” but the salient nature of comparisons with the class mean should lead to “classroom dominance.” The authors also tested whether both standards could be used simultaneously. Using a large sample of primary school students from The Netherlands, they included both predictors in the same model and found only a negative effect of classroom average achievement. A recent study based on Finnish primary found similar classroom-dominance when comparing classroom average and the average of peers students spend time with during and after classes (Koivuhovi et al., 2020). It should be noted that, even though Koivuhovi et al. (2020) used techniques from social network analysis, they did not differentiate social selection and influence mechanics. Furthermore, the study did not examine friendships.

## The Present Study

**Figure 1 fig1:**
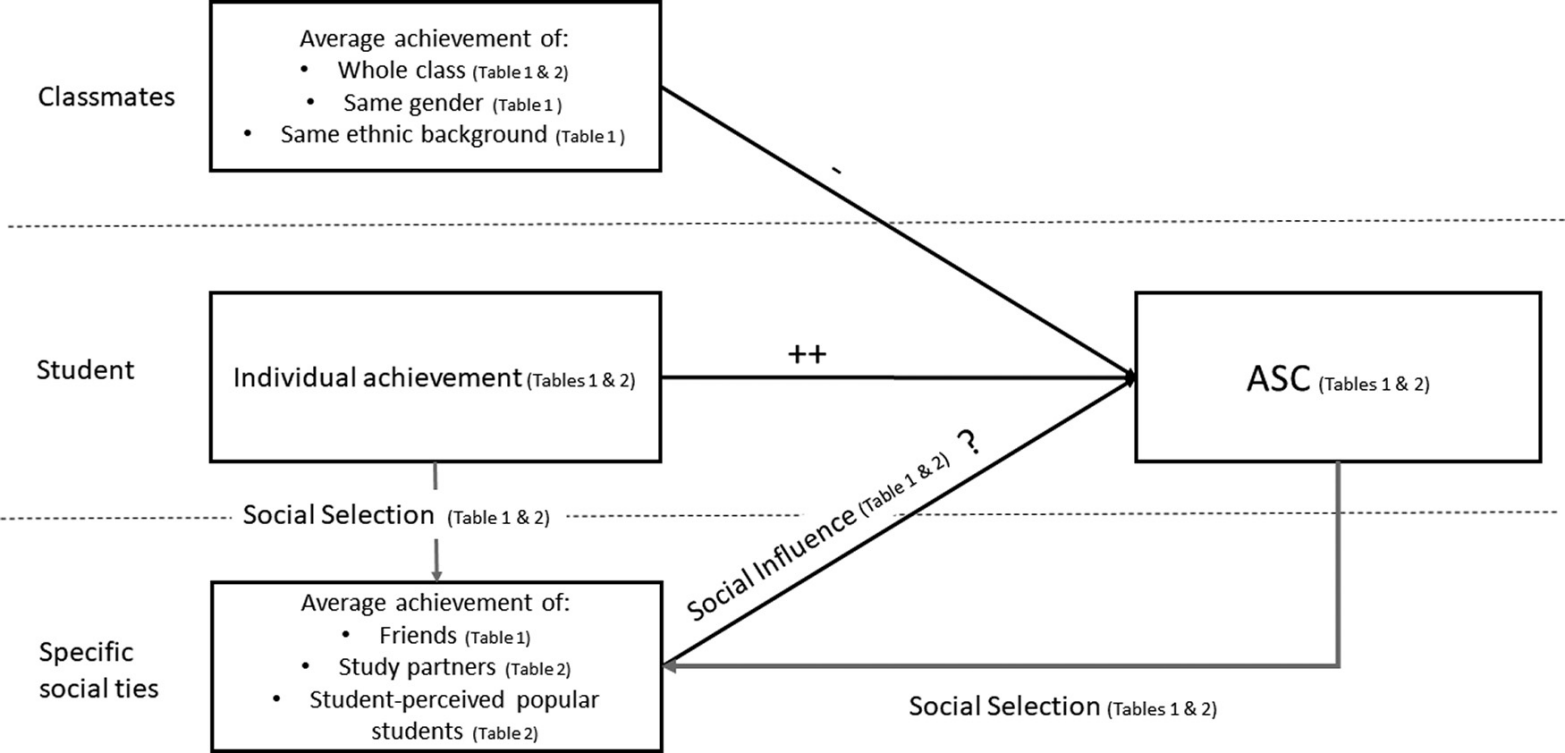
Working Model of our Study Depicting the Possible Effects of Comparisons With Different Peer Groups as Well as Social Selection Mechanisms *Note*. References to the tables that include the models where the different effects are tested are shown in parentheses.

The central research question of this study was how social comparisons with different (overlapping) groups of peers in the classroom affect students’ ASCs. We juxtaposed different frames of reference and peer groups that students might use for their social comparisons using longitudinal social network analysis (SAOMs; Snijders et al., 2010) based on two measurement waves of secondary school students in Germany. Our working model is shown in Figure 1. In addition to the classroom average achievement, we examine friends, study partners, peers the student perceives as popular as well as same-gender and same-ethnic classmates as possible frames of reference.[Fig fig1]

Without considering other frames of reference, we expect the classic BFLPE to be replicated—that is, we expect positive effects of individual achievement on ASC and additional negative effects of *classroom average achievement* (Hypothesis 1).

Regarding the effect of *friend average achievement* on ASC, two conflicting expectations can be formulated. The first argument is based on BFLPE research and the idea of a “local dominance” effect (Koivuhovi et al., 2020): There should be a negative effect of friend-average achievement on ASC indicating contrasting social comparison. In this case, friends would function as a more local frame of reference than the classroom (Zell & Alicke, 2010). In contrast, following arguments made in social psychology, a high similarity between the source and the target of social comparisons should make assimilation effects more likely (Mussweiler, 2003). Thus, a positive effect of friend-average achievement on ASC could be interpreted as an assimilative social comparison effect with students’ ASCs benefiting from high-achieving peers through identification and “basking in reflected glory” (Koivuhovi et al., 2020). On the grounds of previous empirical work on the prevalence of contrasting comparisons, we expect contrast effects be more likely—the effect of friend-average achievement on ASC should thus be negative (Hypothesis 2).

*Study partners* might serve as viable social comparison targets as students know more about their ability and achievement (owing to studying together) than about the achievement of other students. Furthermore, a study group might be perceived as a local frame of reference by the students (Zell & Alicke, 2010). Therefore, as for friends (who substantially overlap with study partners; Stadtfeld et al., 2019), we expect the average achievement of study partner peers to show a negative effect on ASC (Hypothesis 3).

There are no previous studies on social comparison effects with student-perceived popular peers on ASC and it has been shown that popularity cannot easily be inferred from friendship status, but should be assessed separately (Vörös et al., 2019). We expect the achievement of *student-perceived popular peers* to be meaningful for ASC owing to popular peers’ visibility and status (and thus being likely social comparison targets). One argument for assimilation effects could be that students perceived as popular serve as role models for the academic behavior within a class. However, this would only be the case if (a) students think of students they perceive as popular as similar to them in the first place (Mussweiler, 2003), which is less likely than for friends, and/or (b) students have a popularity motive (Jones & Cooke, 2021). Furthermore, contrast effects are generally dominant in most situations (see above). Therefore, we refrain from making a prediction on the direction of social comparison effects with student-perceived popular students and examine whether the effects of the average achievement of student-perceived popular peers on ASC are contrastive/negative (Hypothesis 4.a) or assimilative/positive (Hypothesis 4.b).

Finally, *salient ingroups of same-gender or same-ethnic peers* might constitute a more “local” frame of reference than the class. In that case, students would compare themselves with peers from their ingroups rather than all classmates, but still in a contrasting way. Therefore, the achievement of same-gender and same-ethnic peers should show a negative effect on ASC (Hypothesis 5).

**Figure 2 fig2:**
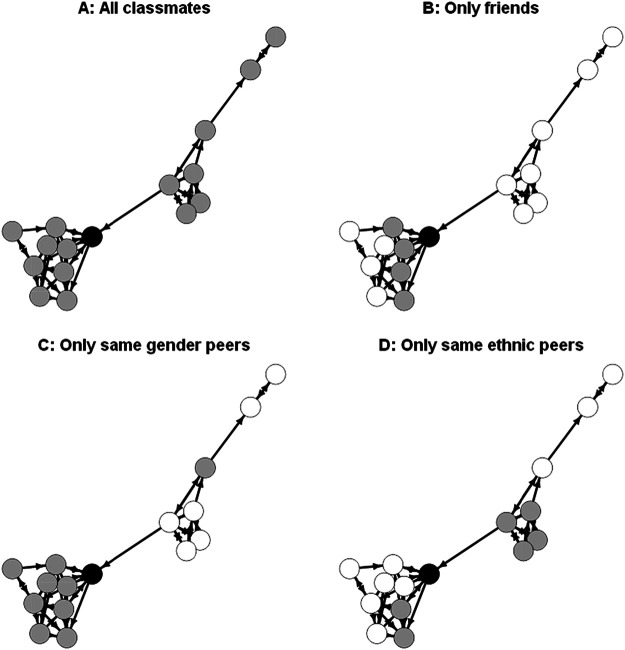
Illustration of Different Possible Comparison Groups Within the Social Network of a Classroom *Note*. Nodes (circles) refer to students, edges (arrows) refer to friendship nominations made by students. The plot was created using the R package igraph. The layout of the nodes is based on the Fruchterman-Reingold algorithm (Fruchterman & Reingold, 1991). The aim of the algorithm is to depict the network based on aesthetic criteria such that the relationship structure (more connected nodes being more central) is well represented and that the visibility of nodes and edges is optimal (e.g., no edges crossing nodes). Black = examplary student, gray = comparison targets, white = classmates not used for comparison. Data from an exemplary classroom in the CILS4SEU dataset.

We tested the effects for specific peers both with and without controlling for classroom average achievement, and our Hypotheses 2 to 5 assume that the effects of specific peers are incremental to possible effects of the classroom average. Through this juxtaposition of different frames of reference (see Figure 2 for an illustration how these different frames of reference can look like in the social network of one example classroom), we could examine whether there is “classroom dominance” or “friendship dominance” (or perhaps dominance of other specific peers) in social comparisons with one frame of reference possibly prevailing (Wouters et al., 2013). We extended the studies by Wouters et al. (2013) and Koivuhovi et al. (2020), who found evidence for “classroom dominance” (i.e., no incremental effect of friend-average achievement beyond a persisting BFLPE) in several ways. First, we separated social selection and influence processes (see below). Second, we considered several types of peers that could be valid comparison targets in addition to friends. Third, both studies were based on primary school samples. However, friendships become much more important as students reach adolescence where peers become their primary social contacts (Larson & Richards, 1991). Therefore, we formulated no hypothesis on the dominance of different social comparison effects. Still, the relative strength and possible coexistence of these effects are essential research objectives of this study.[Fig fig2]

Finally, we note that, using terminology from social network research, the research questions we examine are examples of *social influence*—that is, an individual’s behavior or characteristic (here: students’ ASC) being affected by the behaviors or characteristics (here: achievement) of specific peers such as their friends (Lomi et al., 2011). An old discussion in research on social networks is, however, whether people select friends who are similar to them (social selection based on homophily) or if friends become more similar over time (social influence) or both (McPherson et al., 2001). For the study of social influence processes, it is therefore important to disentangle them from social selection (Cohen-Cole & Fletcher, 2008; Steglich et al., 2010). For example, if students with a higher self-concept would preferably choose friends who also have high self-concepts in a given domain and thereby also have high achievement, this could result in cross-sectional similarities of self-concepts between friends and be misinterpreted as an assimilative social influence effect if selection was not taken into account. Similarly, if students with low self-concept would tend to select students with high achievement as their friends, this could be misinterpreted as a contrastive social influence effect (students with higher achieving friends have lower self-concepts).

Longitudinal social network models such as SAOMs allow us to disentangle social influence and social selection processes (such as homophily). Using such models, homophily regarding gender and ethnicity have been demonstrated to be key criteria for the selection of friends (Lorenz et al., 2021; Raabe et al., 2019), and there is evidence suggesting that academic achievement is a predictor of friendship choices, too, (Gremmen et al., 2017; Laninga-Wijnen et al., 2019). There is much less theoretical and empirical work on the question whereas students’ ASCs could also be linked to friendship selection processes. Identifying as “a good student” and perceiving someone else the same way might be a more important dimension along which to select similar friends than objective achievement. This is supported by empirical work demonstrating similarity among friends regarding self-perceptions and motivational beliefs (Altermatt & Pomerantz, 2003). Another study based on longitudinal social network modeling found that similarity among students regarding academic self-efficacy and achievement can be attributed to selection processes along with self-efficacy and achievement (Shin & Ryan, 2014). To our knowledge, however, no previous studies examined social selection based on ASCs.

## Method

### Dataset

Our study was based on the German sample of the Children of Immigrants Longitudinal Survey in Four European Countries (CILS4EU; Kalter et al., 2019). The CILS4EU is a large-scale panel study of secondary school students with an oversampling of students with immigrant backgrounds and one of the few studies that include sociometric measures (e.g., information about complete social networks within classrooms). The dataset can be acquired for secondary analysis at the research data archive at GESIS | Leibniz Institute for the Social Sciences. Because our study was based on secondary data use, no approval from an ethics committee or institutional review board was necessary. However, data collection for the CILS4EU study in Germany was approved by the ministries for education of the federal states as well as state data protection officers. Our study was not preregistered. Further materials such as analysis code will be made available upon request.

We used the first two measurement waves. The target sample for CILS4EU in Germany were students who attended the 9th grade during the first wave. They were surveyed again 1 year later. Students were selected from all school-types (i.e., lower, intermediate and higher secondary track as well as comprehensive schools; for a more detailed description of Germany’s tracked school system, see Maaz et al., 2008). For the first wave, information regarding 5,013 adolescents’ social networks, self-concepts, school achievements and family backgrounds was collected between October 2010 and March 2011. This base sample size referred to the number of students that were reached and thus the number of cases in the student questionnaire dataset at T1. It resulted from a participation rate of 80.9% on student level. The school and student sampling process, participation rates and fieldwork are described in detail in the technical report (CILS4EU, 2016). The data collection for the second wave (T2) took place about a year later (from September 2011 to February 2012 for 97% of the sample; however, there were a few students for which interviews were conducted later until June 2012).

### Analysis Sample

Our study relied on SOAMs to study social networks longitudinally. These models have particular requirements with regard to the data structure. Thus, following the recommendations in the sociometric fieldwork reports of the CILS4EU (Kruse et al., 2016; Kruse & Jacob, 2016) and previous applications of SAOMs (Boda, 2018, 2019; Lorenz et al., 2020; Raabe et al., 2019), we only analyzed classes with a sufficient participation rate across all waves and without major compositional changes. More specifically, we included classes only if (a) students in those classes participated in the sociometric questionnaire at both measurement points and students did not change the class between the two survey waves (resulting in *N* = 3.858), (b) not more than 25% of students dropped out between the measurement points (resulting in *N* = 2.506) and (c) the class included at least 10 students (resulting in *N* = 2.467). Finally, one class was excluded for which all grade variables were missing.

These exclusions resulted in an analysis sample of 2,438 students from 117 school classes. They were on average 15.18 years old (*SD* = .66) and 47% were male. More than half of the students (56%) had an ethnic minority background with the biggest origin groups being students whose families emigrated from Turkey (19%) and Eastern Europe (10%). Further details on the sample can be found in Supplement 1, Table S1, which includes the sample statistics.

### Measures

#### Social Ties

The CILS4EU study is unique among publicly available panel data sets in its usage of comprehensive sociometric measures. Students were asked to nominate a maximum of five friends from their classroom (“Who are your best friends in class?,” max. five nominations), to report with which students they study together (“Who do you sometimes do your homework with?,” any number of nominations) and who they considered popular (“Who are the most popular students in this class,” max. five nominations). The sociometric questions were included in the student questionnaires at both T1 (9th grade) and T2 (10th grade). Details on the sociometric fieldwork can be found in the technical reports (Kruse et al., 2016; Kruse & Jacob, 2016). In our sample, 30% of friends were also being nominated as study partners and 54% vice versa. Thus, these two sociometric features can be clearly differentiated even though overlap is to be expected (Stadtfeld et al., 2019). Descriptive statistics at the network level (see Supplement 1 for details) showed that the friendship networks were denser than the study partner and perceived-popularity networks. All networks showed sufficient stability between the measurement occasions.

#### Academic Self-Concept

Because there are no domain-specific, multiitem ASC scales in the CILS4EU dataset, we constructed a proxy measure for general ASC. We combined three indicators referring to self-perceived domain-specific achievement in the three main subjects (“How well are you doing in the following subjects?” asked for German, English and mathematics with replies given on a 5-point scale ranging from *not well at all* to *very well*) with two Likert-style indicators of general academic self-efficacy (“I am sure that I can do well at school” and “I am sure that I can get good grades at school” with agreement indicated on a 5-point-scale from *strongly disagree* to *strongly agree*) to create a latent factor. All indicators were assessed in 9th (T1) and 10th (T2) grade. The resulting general ASC factors showed a reasonable, though not ideal fit (which is plausible given its nature as a combined proxy) to the data in a first-order one-factor confirmatory factor analysis (CFA) model (T1: CFI = .921, RMSEA = .091, SRMR = .042, *df* = 5; T2: CFI = .891, RMSEA = .131, SRMR = .057, *df* = 5) as well as high reliability (T1: McDonald’s ω = .82, T2: ω = .86). The model fit could be improved by adding two correlated residuals between the two self-concept measures in the language domains and between the two self-efficacy measures. This led to better model fit (T1: CFI = 1.00, RMSEA = .005, SRMR = .005, *df* = 3; T2: CFI = .999, RMSEA = .015, SRMR = .007, *df* = 3). However, the reliability was lower (T1: ω = .71, T2: ω = .81).

Therefore, factor scores from the CFA models without correlated residuals were used in the further multivariate analysis. Descriptive statistics of the indicators are provided in Supplement 1, Table S1. However, we replicated model 3 for the friendship network using the latent self-concept variable with the two correlated residuals. The results were very consistent across all parameters (see Supplement 5 for details).

#### Academic Achievement

Similar to the procedure for ASC, we computed latent achievement scores based on five indicators that were then used in further analyses. This is in line with the recommendations by Dicke et al. (2018) to use several indicators when computing peer-averages to control for measurement error. We used reverse-scored teacher-assigned grades in German, Mathematics and English (which, in Germany, range from 1 [*excellent*] to 6 [*insufficient*]) as well as the sum scores of a cognitive ability test and a language ability test. Cognitive abilities were measured using a language-free test based on solving figural problems. The language ability tests were country-specific (given that different languages were being tested), but all had a focus on lexical knowledge using synonym- or antonym tests. In Germany, the verbal subscale of the KFT 4–12+ R (Heller & Perleth, 2000) was used which is a well-validated and frequently used ability test for student samples. The achievement tests were only conducted at T1. The latent factor showed a reasonable fit (CFA = .964, RMSEA = .127, SRMR = .028; correlated residuals between the grade indicators were included) and reliability (ω = .67) given that it can be considered a composite achievement measure. We additionally created two separate measures (a) using only the two achievement tests and (b) using only the three grade variables. In a two-factorial CFA model, the fit was adequate (CFI = .964; RMSEA = .090; SRMR = .027) and the latent correlation between the achievement and the grade factor was .49. The results of the main models (model 3 for each of the social networks; see data analysis section) could be replicated with those alternate achievement measures (i.e., separate models for grades and test-scores were estimated), and they were consistent for all hypotheses (see Supplement 4).

#### Further Covariates

*Gender* was self-reported by the students. To assess the *socioeconomic status* (SES) of the students, they were asked to specify the current occupation of their parents. The information was recoded using the International Socio-Economic Index of Occupational Status (ISEI). The ISEI is a continuous measure aiming to classify occupations according to their income, prestige, and the required educational status, ranging from 16 (e.g., unskilled agricultural worker) to 90 (e.g., judge). It is thus a broad measure of SES that taps into parental education, vocational success, and income and has been frequently used in previous studies on social networks based on the same data (e.g., Lorenz et al., 2021). The ISEI was measured for both parents, but the higher ISEI was used in the analyses as an indicator for socioeconomic resources in the family. We further included students’ *ethnic background* which was computed based on the countries of birth of the students, their parents and their grandparents. The classification of origin groups followed the procedure described in Dollmann et al. (2014). In the analyzed sample, it was possible to differentiate between four groups: native majority students (*N* = 1,079), students of Turkish origin (*N* = 454), students of Polish origin (*N* = 254), and students of any other national origin (*N* = 651).

### Data Analysis

#### Stochastic Actor-Oriented Models

SAOMs simultaneously model changes in social networks (e.g., friendship ties) and individual characteristics (e.g., ASCs). SAOMs rely on simulations to infer the social mechanisms that potentially underlie the observed changes in a social network. The technical and mathematical foundations of SAOMs are described in detail in other work (Snijders, 2017; Snijders et al., 2010; Steglich et al., 2010)—in the following we will explain the conceptual idea. The simulations aim to reconstruct the evolution of the observed social network(s) as a sequence of many small changes while taking an actor-oriented perspective. That means actors (e.g., students in a classroom) are assumed to control their outgoing ties (i.e., establishing new friendship ties or maintaining or terminating existing friendship ties) as well as the change in their behavioral characteristics (e.g., increasing or lowering their ASCs). During the simulation process, single actors are randomly selected and given a chance to change a single outgoing social tie or value of a behavioral variable (e.g., ASC). In our case, it is simulated that a single random adolescent creates, maintains, or terminates a friendship tie to one other classmate or changes their ASC by one unit. For this reason, the dependent behavioral variable (here: self-concept) is recoded into a discrete number of categories. In our case, we used quantiles of the distribution and recoded the variable into five categories each representing 20% of the student distribution.

The decisions from the actors in the model are simulated based on effects (independent variables) specified by the researcher. In case of the social ties, the effects represent the rules of tie formation within the network; for the behavioral outcomes, they represent influences of predictors on an individual characteristic comparable to a regression model. Also similar to a regression model, the effects coefficients represent independent marginal effects (each controlling for all other effects) and can be tested for significance individually. The effects can be based on actor attributes (e.g., academic achievement and ASC of the student), the attributes of the other actors in the network (e.g., the average academic achievement of friends), and endogenous network processes (e.g., the reciprocity of friendships). The first survey wave serves as a starting point for simulating the network processes leading to the social network observed during the second wave. Therefore, SAOMs enable the independent study of (a) possible social comparison effects with peers and their effects on ASC (behavioral outcome; this perspective is similar to the classic BFLPE model) and (b) social selection effects based on ASCs.

In the context of SAOMs, each classroom is traditionally considered an independent social network and the simulation processes refer to every single network. This poses the question of how to integrate results from these networks. Recently, a new method became available—random effects stochastic actor oriented models which are implemented using a Bayesian estimate procedure in RSienaTest (SienaBayes, see Ripley et al., 2021). This approach accounts for the hierarchical data structure and multilevel dynamics as it is the case in random-coefficient regression models. This is particularly important because multilevel modeling is the classic framework for testing the BFLPE as it is the effect of a classroom-level (L2) variable (average achievement) on student-level ASC (L1). Using multilevel SAOMs, such L2 effects can also be included and thus the BFLPE and the effect of specific social groups can be included in the same model (see Figure 1). We tested the convergence of our models, as described in Ripley et al. (2021: section 11.3.7). All models achieved sufficient convergence.

#### Model Specification

In a series of multilevel SAOMs, we aimed to identify the effects of social comparisons on students’ ASCs. Thus, ASC was the behavioral outcome variable in all models, whereas the predictors of interest (i.e., interpreted as resulting from social comparisons) were different achievement averages of either all or of specific classmates (see Figures 1 and 2).

As mentioned above, all SAOMs included a *selection model part* (or network dynamics model part) that focuses on social ties and that was used to control selection effects in the estimation of social influence effects. This part of the statistical model was configured congruently across all models. It included structural effects that tap into endogenous network processes (reciprocity, transitive triplets, transitive reciprocated triplets, and three degree effects). In addition, both a selection of actor and target effects (e.g., the effect of a students’ achievement on their tendency to create friendship ties) as well as homophily effects for ASC, achievement and gender, as well as SES and ethnicity were included.

The estimated models varied in the *social influence model part* (or behavior dynamics model) where different predictors of ASC were included. In the first model, in accordance with Hypothesis 1, we aimed to replicate the classic BFLPE using a SAOM framework and thus included students’ individual achievement and the classroom average achievement (Model F.1). Focusing on friendship, we then estimated a model in which friends’ average achievement was included instead of classroom average achievement (Model F.2). Then the effects of the classroom average achievement and the friends’ average achievement were modeled competitively in the same model (Model F.3). Finally, we then added the average achievement of only same-gender classmates (Model F.4) or same-ethnic classmates (Model F.5). Finally, Models 2 and 3 were replicated using study partners (Models S.2 and S.3) and student-perceived popular peers (Models P.2 and P.3) instead of friends. An overview of the models can also be found in Tables 1 and 2, where the results are reported. Furthermore, a detailed description of each effect is included in Supplement 2.[Table tbl1][Table tbl2]

#### Treatment of Missing Data

Missing information in sociometric data poses more severe problems for the reliability and validity of social network analyses compared with missing data in, for example, conventional regression analyses. This is because in network data, when a high proportion of information regarding social ties is missing, key characteristics of the network structure might be misrepresented (e.g., when a central node is missing) which is problematic since the network structure can determine the changes in both social relations among the actors and their behavior (Huisman & Steglich, 2008). Therefore, as described in the “analysis sample” section, we followed the guidelines laid out in the sociometric report of the CILS4EU study (as well as the procedure in previous studies) and only analyzed classes with a sufficient participation rate.

A summary of the missing data in the analysis sample can be found in Supplement 1, Table S1. Owing to the sample restriction procedure, the missing rates for the sociometric indicators are very low at T1 (all < 3.2%) and still relatively low at T2 (< 16.6%). It should be noted that even when students’ nominations were missing (outdegrees), they could still be nominated (indegrees) which is why there are no missing values on the indegree variables. In addition, because the base sample (*N* = 5,013, see above) only includes students that participated in the study at T1 and received instruments (rather than just being contacted), the missing rates were very low for the achievement (< 3%) and the self-concept variables (T1 < .5%; T2 < 8.2%) as well as the control variables, particularly at T1 (see Table S1). The ethnic origin variable is a specific case, where, owing to the oversampling of immigrants in the CILS4EU study, information on immigrant status was also collected from the school administrative data and not just the questionnaire. Therefore, this variable has no missing cases.

At T2, 7.5% of cases were missing for the latent self-concept variable and 13.7% of cases for the friendship outdegree variables. These are thus youth who did not fill out the questionnaires or were not present for testing at T2 (but were still part of the contacted study sample at T2 and could be nominated). Students with missing latent self-concept values at T2 had lower self-concept (*d* = .24, *p* < .01) and achievement (*d* = .53, *p* < .01) at T1. Students with missing sociometric data at T2 also had lower self-concept (*d* = .28, *p* < .01) and achievement (*d* = .33, *p* < .01) at T1. It is plausible that these youth were indeed less engaged at school and thus not present on testing day at T2. However, given the low percentage of missing data overall, we do not think this could influence the results. Furthermore, no students were dropped from the sample using listwise deletion. Rather, within the network simulation models, missing values were imputed for both the dependent variables and covariates as described by Ripley et al. (2021: section 4.3.2).

## Results

### Social Selection Model

In the following, we report results of the SAOMs. A discussion of descriptive statistics for all variables, bivariate correlations as well as network level descriptive statistics can be found in Supplement 1. We briefly report the results from the social selection part of the model first because it serves to disentangle these processes from the social influence processes that we are theoretically interested in.

Social selection processes based on individual characteristics can either be actor (or ego) effects (e.g., students with higher achievement making more friendship nominations), target (or alter) effects (e.g., students with higher achievement being nominated more frequently), or homophily effects (e.g., students nominating other students with similar achievement to themselves as friends). The social selection part included the same predictors in each model—thus, the models only differed in the behavior dynamics part (predictors of ASC) and whether friendship (see Table 1) or costudying/perceived popularity (see Table 2) were used as the social network. The parameter estimated can be interpreted similarly to conditional log odds rations in logistic regression models (see Chapter 13 in Ripley at al., 2021). Aside from the structural network effects (see Supplement 2 for an overview of all effects that were modeled and Supplement 3 for all parameters), there were several significant ego, alter, and homophily effects. For example, in terms of friendship, female students nominated fewer peers as friends (B = −.11, *p* < .05; parameter from Table 1, model F.1), but were nominated more often (B = .06, *p* < .05). Also, we observed strong gender homophily (B = .30, *p* < .05). Furthermore, there were significant homophily effects of both achievement (B = .46, *p* < .05) and ASC (B = .15, *p* < .05) for friendship selection. In the costudying network, we found homophily effects for gender, social background, ethnic background, and achievement similar students along these dimensions were more often reported as study partners (see Table 2, Model S.2). In the popularity network, there were fewer significant effects in the selection model, but we also observed a homophily effect for gender (i.e., students are more likely to name students from their own gender as popular; B = .34, *p* < .05; see Table 2, Model P.2). Overall, the social selection models show that similarities in individual attributes contribute to not only the selection of friends and study partners, but also whom one perceives as popular. Importantly, SAOMs allow us to estimate social influence, presented in the next sections, while controlling for these selection processes.

### Social Comparisons With the Class Average: Replicating the BFLPE (Hypothesis 1)

The classic BFLPE is represented by a positive effect of individual achievement and an incremental negative effect of class-average achievement on ASC. As the BFLPE affects students’ ASCs, the coefficients of interest can be found in the behavior dynamics part of the SAOM depicted in Table 1, Model F.1. This model provides significant evidence for the BFLPE: students with higher achievement show a more positive ASC (B = .31, *p* < .05), but, given similar individual achievement, students in higher-achieving classes show lower ASCs (B = −.23, *p* < .05). Thus, the BFLPE could be replicated and its classic interpretation would suggest that students make contrasting social comparisons with the class-average.

### Social Comparisons With Friends, Study Partners, and Student-Perceived Popular Peers (Hypotheses 2 to 4)

In the next model (Table 1, Model F.2), we included the average achievement of friends instead of class-average achievement as a predictor of ASCs. We found a similar pattern of effects with a positive individual effect of achievement on ASC (B = .26, *p* < .05) and a negative effect of friend-average achievement (B = −.10, *p* < .05). Interpreted similarly to the BFLPE, this would mean that students make contrasting social comparisons with their friends (evidence for Hypothesis 2). We also estimated this model using study partners and student-perceived popular peers (see Table 2, Models S.2 and P.2) rather than friendship to define the social ties and the achievement aggregates. For student-perceived popular peers, we also found a negative contrast effect (B = −.16, *p* < .05), which would speak in favor of Hypothesis 4.a (and thus against 4.b). For study partners, we did not find a significant effect (no evidence for Hypothesis 3).

After first investigating the effects of friends, study partners, and student-perceived popular peers without taking into account the average class achievement, we juxtaposed the frames of references in the next step to examine the relative strength of the social comparison effects (friendship vs. classroom dominance) as well as possible incremental and suppressor effects. In Table 1, Model F.3, we included both the effects of all classmates’ and friends’ average achievement. There was a negative effect of average classroom achievement (i.e., the BFLPE) which was nearly as strong as in model 1 (B = −.21, *p* < .05), but we no longer saw a significant effect of average friend achievement (B = .03, *p* = .25). These results thus speak for “classroom dominance” (Wouters et al., 2013) and no longer provide evidence for Hypothesis 2. We found similar results (i.e., a persistent negative effect of average classroom achievement) when examining study partners and student-perceived popular peers (Table 2, Models S.3 and P.3). Interestingly, however, there seems to be a beneficial effect of studying with high-achieving peers for students’ ASCs (see Table 2, Model S.3). This could be interpreted as an assimilation or “reflected glory” effect (i.e., students moving their self-evaluations toward the achievement of their study-peers) and goes against the expected contrast effect we formulated in Hypothesis 3. However, it should be noted that the effect is small and failed to reach significance in the two models estimated for robustness using only grades and only test-scores (probably for reasons of power resulting from a narrower achievement factor; see Supplement 4). In contrast, the average achievement of student-perceived popular peers did not show a significant effect (no evidence for Hypotheses 4.a and 4.b after controlling for average classroom achievement).

### The Role of Same-Gender and Same-Ethnic Classmates (Hypothesis 5)

In the final step, we added the average achievement of same-gender and same-ethnic classmates to see whether students compare themselves primarily with peers from specific ingroups. We found no incremental effects comparisons with these two social ingroups and the same patterns of a persistent negative effect of the classroom average (see Table 1, Models F.4 and F.5; additional support for Hypothesis 1, no support for Hypothesis 5 as an incremental effect).

## Discussion

In this study, we juxtaposed several frames of reference that students might use for social comparisons to adapt their ASCs. Thereby, we aimed to bridge (a) research on the BFLPE emphasizing the importance of the classroom average and (b) works from social psychology that had pointed out that the subjective relevance of peers should matter for their selection as social comparison targets—thus far, these lines of research were largely unconnected (Huguet et al., 2009; Wouters et al., 2013). We replicated the classic BFLPE—a negative contrast effect of average classroom achievement on individual ASC—using longitudinal social network analysis as the first study to apply such models in research on ASC (Hypothesis 1). In models that did not consider the classic BELPE, we found the average achievement of friends and student-perceived popular peers to show contrast effects on ASC as well. However, these effects disappeared when classroom average achievement was controlled in the models. That is, in models that simultaneously included the average achievement in the classroom and the average achievement of specific types of peers, only the former exerted a significant influence on students’ ASC. In these models, we observed no additional contrast or assimilation effects of friends or student-perceived popular peers (evidence against Hypotheses 2, 4a and 4b). Only for study partners, we found an assimilation rather than a contrast effect once classroom average achievement was controlled for (contrary to Hypothesis 3). That is, students’ ASC seemed to have benefited from studying with higher-achieving peers—though this effect was not robust. The average achievement of same-gender and same-ethnic peers did not show incremental effects (Hypothesis 5 rejected).

Our Hypotheses 2 to 5 regarding the role of specific peer groups were based on insights from experimental social comparison research: Research on the selection of social comparison targets suggested that, for example, the similarity to the social comparison target or availability of information might play a role (Festinger, 1954; Gerber et al., 2018; Mussweiler, 2003; Mussweiler & Rüter, 2003). We could not find evidence for these hypotheses in the classroom setting. Rather, our main finding is a tendency for “classroom dominance” (Wouters et al., 2013) rather than “local dominance” (Zell & Alicke, 2010). This indicates that the average achievement of the whole classroom seems to exhibit the strongest social comparison effect on students’ ASC. At the same time, the average achievements of specific peers/subgroups of peers such as friends, study partners peers, student-perceived popular peers, and salient social ingroups based on gender and ethnicity seem to be less influential (or do not even exert any influence at all). In that sense, our results are very similar to those reported by other studies (Koivuhovi et al., 2020; Wouters et al., 2013). However, unlike these studies, we considered social selection processes and examined different subgroups of peers (such as study partners and student-perceived popular peers). In this way, our study provides new evidence that adds to a consistent overall picture.

### Implications for Social Comparison Theory

In general, people actively select their friends and their social peer group (McPherson et al., 2001) and, in that same sense, people are assumed to choose their social comparison targets based on the target peers that are available for social comparisons, the motives for social comparisons (e.g., information, self-enhancement, protection against self-worth threat etc.), the characteristic that is compared, and other aspects (Festinger, 1954; Gerber et al., 2018; Lubbers et al., 2009). However, even though there are strong arguments from social psychology, for instance based on similarity, availability of information, and peer status, that would speak in favor of a more important role of specific peer subgroups, the present study shows that class-average exhibits the strongest effect.

We argue that the BFLPE is so dominant in our study and, as shown in other studies, also very universal (Seaton et al., 2009) compared with other social comparison situations (e.g., social comparisons concerning nonformal abilities and characteristics such as personality aspects) because the classroom situation is unique. Students do not actively select their classmates. Owing to the nature of this “mandatory” social setting, social comparisons may become very salient and easily triggered for students. School is all about fostering skills and abilities, and these skills and abilities become visible for classmates as soon as students engage with the learning content. For instance, teachers give positive or negative performance feedback to individual students in attendance of all other students during classes. Sometimes, grades are given “on a curve” and thus partly represent class rank. In this setting, adolescents have less freedom to choose (a) who they want to be with and (b) who to compare themselves to (because the teacher might, in any case, use social comparisons). These conditions seem to lay the ground for social comparisons to the class average (the “generalized other”) and for trying to estimate one’s rank in the class rather than to discount nonsimilar students when making comparisons. A similar argument has been made by Diener and Fujita (1997) as well as by Dai and Rinn (2008), who differentiate between “imposed” and “self-engendered” social comparisons (p. 290) pointing out that most of the literature within the field of social psychology focuses on active comparison process, that include target selections, whereas the BFLPE is seen as situationally imposed. Furthermore, it could be argued that the class-mean provides more diagnostic value than comparisons with individual classmates and is thus a more valid comparison target (Wouters et al., 2013)—in that sense, it may even be the most “rational” comparison standard for students to choose. After controlling for classroom average achievement, we found a tendency for small assimilation effects of the achievement of study partners. These seem to coexist with the contrasting social comparisons made with the classroom average. This result would be consistent with studies showing that comparisons with the class average and with individual peers can coexist (Huguet et al., 2009).

### Limitations and Open Questions

We used latent factors with several indicators as measures for both general ASC and achievement. We combined test scores and grades and argued that this provides the best estimate of the overall peer performance as researchers have previously warned that using just one indicator of peer-average performance can lead to “phantom effects” owing to measurement error (Dicke et al., 2018). Still, it can be argued that the proxy indicators are not ideal: Test scores and grades capture different aspects of achievement, and ASC is considered a domain-specific construct (even though general ASC is also commonly studied). It is a well-known finding in research on ASC that the relation between ASC and achievement is higher when studying it on a domain-specific level as opposed to a general level (Valentine et al., 2004). Accordingly, the BFLPE also seems to be a bit smaller for general ASC than domain-specific ASC (Fang et al., 2018). Thus, we would expect social comparison effects to be stronger on domain-specific measures. Furthermore, there are conceptual differences between ASC and self-efficacy (Bong & Skaalvik, 2003; Jansen et al., 2015), even though the items that were used to measure general school-related self-efficacy were close in wording to typical self-concept items. Ideally, data sets should include both a multiitem domain-specific ASC scale and scores from domain-specific achievement test. However, we do not know any available dataset which includes such measures in combination with information on students’ social networks. Given these constraints, we deem the latent factor approach as preferable over using single-item indicators.

Germany has a relatively strong between-school tracking system in secondary school. After four to six years (depending on the federal state) years of primary school, students are tracked into different school types. Traditionally there was a vocational (Hauptschule), an intermediate (Realschule), and an academic track (Gymnasium), which was also the case in most states during the time of testing. During the last years, comprehensive schools became more common, and in many states, there are now only two tracks, but there is still an important distinction between academic track and nonacademic track schools. Once in a track, students do not move between classes until grade 11 (when course-by-course teaching starts). Thus, we can generally expect students from a class to show some similarity in achievement, leading to a relatively higher between-school variance in tracked education systems than in untracked/integrated systems. Earlier studies have provided evidence for the BFLPE in school systems with and without between-school tracking, also numerous times in Germany (Chmielewski et al., 2013; Fang et al., 2018; Marsh et al., 2018; von Keyserlingk et al., 2019). We, therefore, know that social comparisons also take place in tracked education systems. However, besides possible differences in the between-school variance in average class achievement, we do not expect social comparisons with the classroom to operate differently across tracked and untracked education systems (e.g., other countries or younger student cohorts). Still, it could be the case that more substantial heterogeneity of achievement within a class would lead to students building more distinct social networks in terms of how those networks are composed. Consequently, social comparisons with particular peers might matter more in such contexts than in contexts with strong between-school tracking. It would indeed be interesting to replicate the study results in other education systems.

Furthermore, we found a high correlation between average classroom achievement and the average achievement of social ingroups. When there are no clear achievement differences between student groups, these effects are possibly confounded and difficult to disentangle. Still, our random effects SAOMs produced effect estimates with reasonable standard errors and we found significant effects even though there was a substantial correlation between the predictors. These effects indicated a dominance of classroom average achievement with no additional effects of same-gender or same-ethnicity average achievement, and this result is substantially in line with both the theory and the results of our other models.

Finally, our study produced results on friendship selection that might inspire future research in developmental and educational psychology. Students select their friends based on their own characteristics (ego), the characteristics of their peers (alters), and dyadic characteristics such as similarity in these attributes. Previous studies have shown such processes for sociodemographic characteristics such as gender and ethnicity, but also academic achievement (Gremmen et al., 2017; Lorenz et al., 2020, 2021). We replicated those effects. In addition, we found similarity effects based on ASC which had not been tested in previous studies. Thus, students cluster in friendship cliques with similar ASCs and such a clustering appears independently of the clustering along with achievement and sociodemographic characteristics such as gender and ethnicity. Future work could aim to replicate this explorative result and to look into the mechanisms and possible theoretical explanations for these selection effects. It is conceivable that ASC, as an important part of general self-concept and an aspect of identity (the image of being or not being “a good student”), might be just as important and visible for peers as the achievement itself.

It would also be valuable to investigate whether the similarity in domain-specific ASCs and other domain-specific motivational characteristics such as interest drive the selection of specific peers such as friends. This person-peer/group similarity judgment as well the extent to which students attain to popularity goals (Jones & Cooke, 2021) are also important for possible social comparisons with student-perceived popular peers. Future studies could aim to assess these additional constructs to better understand under which conditions social comparisons with friends, study partners, and student-perceived popular students might be made. Still, it seems unlikely to find subgroups (with particular motives or configurations of similarity) for which the BFLPE is not still more dominant than assimilation effects given the strong evidence for the universality of the BFLPE (Marsh et al., 2021).

Overall, our study shows the importance of the classroom context as a “total environment” for social comparisons (Diener & Fujita, 1997). Compared with the salience of using comparisons to find out one's own within-class rank, social comparisons with specific groups of peers such as friends pale in importance. A practical implication could be for teachers to be aware of social comparison processes, the BFLPE, and in particular of the role of “classroom dominance”—that is, to know that students seem to be very well aware of their within-class rank and that this rank is a central source of their ASCs.

## Supplementary Material

10.1037/dev0001368.supp

## Figures and Tables

**Table 1 tbl1:** SAOMs Predicting Changes in Friendship Status (Network Dynamics) and Academic Self-Concept (Behavior Dynamics)

	Model F.1		Model F.2		Model F.3		Model F.4		Model F.5	
Measure	Est.	95% CI	Est.	95% CI	Est.	95% CI	Est.	95% CI	Est.	95% CI
Network dynamics: Tie selection										
Structural network effects (reciprocity, transitive triplets, transitive reciprocal triplets, outdegree: activity, outdegree:density, indegree: popularity)		x		x		x		x		x
Gender: ego	**−0.11**	[−0.18, −0.04]	**−0.11**	[−0.19, −0.05]	**−0.11**	[−0.17, −0.05]	**−0.11**	[−0.18, −0.04]	**−0.10**	[−0.18, −0.02]
Gender: alter	**0.06**	[0, 0.11]	**0.06**	[0, 0.11]	**0.06**	[−0.01, 0.12]	**0.06**	[−0.01, 0.12]	**0.05**	[−0.01, 0.12]
Gender homophily (same gender)	**0.30**	[0.25, 0.36]	**0.30**	[0.25, 0.36]	**0.31**	[0.24, 0.36]	**0.31**	[0.25, 0.36]	**0.30**	[0.23, 0.36]
SES homophily (similarity)	0.00	[−0.11, 0.13]	0.00	[−0.12, 0.12]	0.02	[−0.11, 0.12]	0.00	[−0.13, 0.12]	0.00	[−0.12, 0.11]
Ethnic homophily (same ethnic background)	**0.12**	[0.08, 0.17]	**0.12**	[0.07, 0.16]	**0.12**	[0.08, 0.17]	**0.12**	[0.07, 0.18]	**0.12**	[0.07, 0.17]
Achievement ego	0.03	[−0.01, 0.07]	**0.03**	[0, 0.07]	0.03	[−0.01, 0.07]	**0.03**	[−0.01, 0.07]	0.03	[−0.01, 0.07]
Achievement alter	**0.06**	[0.03, 0.1]	**0.07**	[0.03, 0.1]	**0.06**	[0.03, 0.1]	**0.06**	[0.03, 0.1]	**0.06**	[0.03, 0.1]
Achievement homophily (similarity)	**0.46**	[0.24, 0.7]	**0.45**	[0.24, 0.66]	**0.45**	[0.23, 0.69]	**0.46**	[0.27, 0.67]	**0.45**	[0.21, 0.66]
ASC ego	0.02	[−0.01, 0.05]	0.02	[−0.01, 0.05]	0.01	[−0.01, 0.04]	0.02	[−0.01, 0.04]	0.02	[−0.01, 0.04]
ASC alter	0.00	[−0.03, 0.02]	0.00	[−0.03, 0.02]	0.00	[−0.02, 0.02]	0.00	[−0.03, 0.03]	0.00	[−0.02, 0.02]
ASC homophily (similarity)	**0.15**	[0.02, 0.27]	**0.16**	[0.03, 0.3]	**0.14**	[0.01, 0.29]	**0.15**	[0.01, 0.29]	**0.16**	[0.04, 0.29]
Behavior dynamics: Academic self-concept										
Linear and quadratic shape effect		x		x		x		x		x
Main effect of gender, SES and ethnic origin		x		x		x		x		x
Individual achievement	**0.31**	[0.26, 0.38]	**0.26**	[0.21, 0.31]	**0.28**	[0.24, 0.32]	**0.28**	[0.23, 0.34]	**0.28**	[0.24, 0.33]
Av. achievement of whole classroom (BFLPE)	**−0.23**	[−0.31, −0.15]			**−0.21**	[−0.37, −0.11]	**−0.17**	[−0.39, 0.03]	**−0.16**	[−0.29, −0.04]
Av. achievement of nominated peers friends			−0.10	[−0.16, −0.03]	0.03	[−0.07, 0.14]	0.06	[−0.05, 0.17]	0.05	[−0.05, 0.14]
Av. achievement of same-gender classmates							−0.06	[−0.24, 0.12]		
Av. achievement of same-ethnic classmates									−0.07	[−0.19, 0.04]
*Note*. Bold print = significant (*p* < .05). ASC = academic self-concept; BFLPE = Big-Fish-Little-Pond Effect; SES = socioeconomic status.										

**Table 2 tbl2:** SAOMs Predicting the Selection of study Partners (S) and the Perception of Popular Peers (P) as Well as Academic Self-Concepts (Behavior Dynamics)

	Model S.2		Model S.3		Model P.2		Model P.3	
Measure	Est.	95% CI	Est.	95% CI	Est.	95% CI	Est.	95% CI
Network dynamics: Tie selection								
Structural network effects (reciprocity. transitive triplets. transitive reciprocal triplets. outdegree: activity. outdegree:density. indegree: popularity)		x		x		x		x
Gender: ego	0.03	[−0.07, 0.12]	0.03	[−0.05, 0.11]	−0.03	[−0.08, 0.02]	−0.03	[−0.08, 0.01]
Gender: alter	0.05	[−0.04, 0.13]	0.05	[−0.05, 0.14]	**−0.07**	[−0.12, −0.03]	**−0.08**	[−0.13, −0.03]
Gender homophily (same gender)	**0.68**	[0.62, 0.75]	**0.68**	[0.59, 0.75]	**0.34**	[0.29, 0.39]	**0.34**	[0.29, 0.39]
SES homophily (similarity)	**0.14**	[−0.03, 0.3]	0.14	[−0.03, 0.33]	0.10	[−0.03, 0.22]	0.08	[−0.04, 0.22]
Ethnic homophily (same ethnic background)	**0.19**	[0.12, 0.26]	**0.20**	[0.14, 0.26]	**0.06**	[0.01, 0.11]	**0.06**	[0.01, 0.11]
Achievement ego	**−0.06**	[−0.1, −0.02]	**−0.07**	[−0.11, −0.02]	**−0.04**	[−0.07, −0.01]	**−0.04**	[−0.07, −0.01]
Achievement alter	**0.08**	[0.03, 0.12]	**0.06**	[0.02, 0.11]	0.01	[−0.02, 0.04]	0.01	[−0.02, 0.04]
Achievement similarity	**0.46**	[0.11, 0.78]	**0.47**	[0.12, 0.74]	0.17	[−0.08, 0.42]	0.16	[−0.06, 0.38]
ASC ego	**0.08**	[0.05, 0.12]	**0.08**	[0.05, 0.12]	0.02	[−0.01, 0.05]	0.02	[−0.01, 0.04]
ASC alter	0.01	[−0.02, 0.05]	0.02	[−0.02, 0.05]	**0.02**	[0, 0.05]	**0.02**	[0, 0.04]
ASC similarity	0.16	[−0.04, 0.33]	0.11	[−0.08, 0.29]	−0.01	[−0.14, 0.12]	−0.02	[−0.15, 0.12]
								
Behavior dynamics: Academic self-concept								
Linear and quadratic shape effect		x		x		x		x
Main effects of gender. SES and ethnic origin		x		x		x		x
Individual achievement	**0.25**	[0.21, 0.29]	**0.28**	[0.24, 0.33]	**0.27**	[0.22, 0.32]	**0.28**	[0.24, 0.32]
Av. achievement of whole classroom (BFLPE)			**−0.25**	[−0.34, −0.15]			**−0.16**	[−0.27, −0.01]
Av. achievement of nominated peers:								
Study partners	−0.05	[−0.12, 0.03]	**0.11**	[0.02, 0.2]				
Popular peers					**−0.16**	[−0.24, −0.09]	−0.02	[−0.17, 0.09]
*Note*. Bold print = significant (*p* < .05). ASC = academic self-concept; BFLPE = Big-Fish-Little-Pond Effect; SES = socioeconomic status.								

## Footnotes

1 The term “target” has been inconsistently used in the literature on social comparisons. Some authors (e.g., Mussweiler, 2003) use (evaluation) “target” to refer to a person that is being evaluated against a comparison standard. This usage is mostly common in experimental work where the participants are asked to evaluate different persons with regard to different standards. In many other studies, “social comparison target” refers to another person or group of persons that is chosen by the person making the social comparison as a standard to compare oneself with (i.e., the target is the comparison standard). Following the recent meta-analysis by Gerber et al. (2018), we use this terminology. Thus, in this study, the social comparison target refers to a classmate or group of classmates with whom the students compare themselves.
