# Supplementary material for: Social Comparison Effects on Academic Self-Concepts—Which Peers Matter Most?
Source: Dev Psychol. 2022 Apr 25;58(8):1541–56. doi: 10.1037/dev0001368 (PMC9281383; doi:10.1037/dev0001368)
Supplement: Supplementary file 1 [file DEV-2021-4100_Supplemental_Materials.pdf]

Social comparison effects on academic self-concepts – Which peers matter most?

Online Supplementary Material

Supplement 1: Descriptive Statistics

Descriptive statistics for all variables we included can be found in Table S1. There was a small tendency towards ceiling effects for the self-efficacy items that were used as indicators for ASC. Still, given they only represent two of the five factor indicators, the factor scores were reasonably close to a normal distribution. There were no ceiling or floor effects for the achievement variables.

A common indicator used when working with sociometric items are the number of outgoing social ties of a student (his/her outdegree; e.g., those whom a student nominates as friends) and the number of incoming ties (his/her indegree; e.g., those by whom a student is nominated). At the first measurement point, students nominated an average of 3.46 friends (outdegree) and were nominated an average of 3.80 times (indegree). For peers that students "sometimes do homework with", both the outdegree and the indegree were much lower indicating that most students did not regularly do homework with a lot of students. Quite plausibly, the indegrees for the popularity nominations showed a higher variance and range than the other two sociometric indicators (see Vörös et al., 2019). Table S2 shows the correlations between the different achievement aggregates. As expected, the correlations were substantial. For example, the correlations between classroom achievement and the specific achievement averages of friends, study partners, and popular students were in the range of r = .56-.62. Both same-gender and same-ethnicity achievement, quite plausibly, correlated highly with the classroom average achievement. As expected, the correlations were substantial. For example, the correlations between classroom achievement and the specific achievement averages of friends, study partners, and popular students were in the range of r = .56-.62. Both same-gender and same-ethnicity achievement, quite plausibly, correlated highly with the classroom average achievement. In addition, the correlations between the

achievement of friends, study partners, and students perceived as popular are quite high. This is partly because of between-school tracking, leading to a substantial similarity in classmates' achievement. In addition, as described in the theoretical background section, we would expect an overlap between these groups (e.g., friends becoming study partners and vice versa). Still, as described in the method section, the overlap between the groups is only partial.

Table S3 in the supplemental material shows some descriptive statistics on the network level. The density of a social network is defined by the number of ties that are present in a network vs. the possible number of ties in the network (if every student was tied to each other). It is higher for friendship and popularity than for co-studying. Also, classrooms vary in the density of their networks with regard to all three characteristics. The Jaccard index describes the stability of social networks across time by showing the percentage of ties that are present in both network states within all ties that exist at least once (0 = no ties are present in both, i.e. no stability; 1 = all ties are present in both, i.e. no changes). On average, the friendship networks were a bit more stable than the co-stuyding and popularity networks. The latter two also showed a stronger variation between classes in stability which is likely because our analysis sample was chosen based on a minimum stability of friendship networks—as these are our central sociometric variable and typically include more ties than the other two—while not using the stability of co-stuyding and popularity networks as criteria for sample selection.

Table S1

Descriptive Statistics for Student Characteristics

**Variable**	**%**	**M**	**SD**	**Min.**	**Max.**	**Miss. %**
**Academic self-concept T1**						
Indicators of the latent factor:						
Self-perceived performance German		3.48	0.75	1	5	0.2%
Self-perceived performance Mathematics		3.30	1.00	1	5	0.1%
Self-perceived performance English		3.32	0.95	1	5	0.3%
Self-Efficacy 1 (“I am sure that I can do well at		4.50	0.57	1	5	0.2%
school”)		4.41	0.59	1	5	
Self-Efficacy 2 (“I am sure that I can get good grades						0.5%
at school”)						
Latent factor overall						0.0%
**Academic self-concept T2**						
Indicators of the latent factor:						
Self-perceived performance German		3.53	0.76	1	5	7.7%
Self-perceived performance Mathematics		3.37	1.02	1	5	7.8%
Self-perceived performance English		3.40	0.93	1	5	7.8%
Self-Efficacy 1 (“I am sure that I can do well at		4.64	0.56	1	5	8.0%
school”)		4.45	0.66	1	5	
Self-Efficacy 2 (“I am sure that I can get good grades						8.2%
at school”)						
Latent factor overall						7.5%
**Academic Achievement T1**						
Indicators of the latent factor:						
Grade German		2.94	0.85	1	6	3.0%
Grade Mathematics		3.09	1.03	1	6	2.7%
Grade English		3.06	0.93	1	6	3.0%
Cognitive Ability Test (Sum-Score)		19.45	3.99	1	27	0.3%
Language Test (Sum-Score)		11.85	4.54	1	24	0.2%
Latent factor overall						0.0%
**Social Ties T1**						
Friendship Indegrees		3.76	2.00	0	11	0.0%
Friendship Outdegrees		3.46	1.35	0	5	0.7%
Study partners indegrees		1.25	1.31	0	9	0.0%
Study partners outdegrees		1.18	1.47	0	25	3.0%
Perceived popularity indegrees		2.59	3.52	0	20	0.0%
Perceived popularity outdegrees		2.51	1.72	0	5	3.2%
**Social Ties T2**						
Friendship Indegrees		3.10	1.96	0	8.00	0.0%
Friendship Outdegrees		3.27	1.36	0	5.00	13.7%
Study partners indegrees		1.34	1.51	0	6.00	0.0%
Study partners outdegrees		1.46	2.54	0	28.00	16.6%
Perceived popularity indegrees		1.34	1.51	0	15.00	0.0%
Perceived popularity outdegrees		1.46	2.54	0	5.00	16.6%
Gender						
female	53%					
male	47%					
SES (higher ISEI index of parents)		47.25	20.12	14.21	88.70	6%
Ethnic Background						0%
Majority (German)	44%					
Turkey	19%					
Eastern Europe	10%					
Other	27%					

Note. % = frequencies for categorical variables (of valid cases), Miss % = proportion of missing values. Grades in Germany range from 1 (excellent) to 6 (insufficient). They are represented in their original metric here but were recoded for the construction of the latent achievement factor. If the students didn’t give any answer on any

item of the language skills or cognitive ability test, the whole test was coded as missing. The self-efficacy items are coded from 1 (“strongly disagree”) to 5 (“strongly agree”). Subjective performance was coded from 1 (“not well at all”) to 5 (“very well”). All latent factors were then standardized before the analysis. All statistics are based on the analysis sample (N = 2,438); the selection of the analysis sample is described in the manuscript.

Table S2

Correlation matrix

Variable	1	2	3	4	5	6	7	8
1. ASC T1								
2. ASC T2	.32**							
3. Individual ACH	.05*	.19**						
4. Average ACH: classroom	.00	.04	.67**					
5. Average ACH: friends	.00	.05*	.62**	.86**				
6. Average ACH: study partners	.02	.08**	.56**	.74**	.84**			
7. Average ACH: popular students	-.01	.01	.56**	.83**	.80**	.70**		
8. Average ACH: same-gender classmates	-.00	.02	.62**	.96**	.86**	.76**	.81**	
9. Average ACH: same-ethnic classmates	-.02	.02	.62**	.89**	.80**	.70**	.76**	.85**

Note. ASC = academic self-concept (latent factor), ACH = Achievement (latent factor; only measured at T1)
* $p < .05$.
** $p < .01$.

Table S3

Descriptive Statistics for Classroom Networks (N = 117 networks)

	M	min	max	*SD*
Size	20.84	10	31	5.28
**Friendship**				
Density (t1)	0.26	0.14	0.58	0.08
Density (t2)	0.20	0.11	0.47	0.06
No. of ties (t1)	78.39	19	142	25.86
No. of ties (t2)	64.58	15	115	23.19
Jaccard index	0.46	0.26	0.75	0.09
**Co-Studying (Homework)**				
Density (t1)	0.10	0.01	0.30	0.06
Density (t2)	0.10	0.01	0.41	0.07
No. of ties (t1)	25.94	0	89	16.36
No. of ties (t2)	27.97	1	168	23.84
Jaccard index	0.30	0.03	0.98	0.19
**Popularity**				
Density (t1)	0.25	0.10	0.54	0.09
Density (t2)	0.17	0.03	0.44	0.08
No. of ties (t1)	53.97	13	107	20.54
No. of ties (t2)	40.68	7	85	18.51
Jaccard index	0.27	0.03	0.60	0.11

Supplement 2: Details of the model specification and description of effects

**Effect name (internal RSiena effect name)**	**The tendency is modelled…**
Dependent variable: friendship / co-study peers / perceived popularity (ego creates or maintains a tie to alter)	
Outdegree (density)	To create and maintain ties
Reciprocity (recip)	To reciprocate friendships
Transitive triplets (transTrip)	To create and maintain ties to friends-of-friends (or to those perceived as popular by those one perceives as popular / to those one's study partners report to study with)
Transitive reciprocated triplets (transTrip)	To create reciprocated ties to friends-of-friends (or to those perceived as popular by those one perceives as popular / to those one's study partners report to study with)
Outdegree activity (outAct)	For egos with high outdegrees to create and maintain more friendships
Indegree popularity (inPop)	To create and maintain ties to actors who already have more incoming ties in the network
Gender: ego (egoX)	For girls to create and maintain more ties
Gender: alter (alterX)	To create and maintain more ties to girls than to boys
Gender: homophily (sameX)	For actors to be tied to those of the same gender
SES homophily (simX)	For actors to be tied to those of a similar SES
Ethnic homophily (sameX)	For actors to be tied to those with the same ethnic origin (based on four categories as mentioned in the method section)
Achievement ego (egoX)	For students with higher achievement to create and maintain more ties
Achievement alter (altX)	To create and maintain more ties to students with higher achievement
Achivement homophily (simX)	For actors to be tied to those with similar achievement
Self-concept ego (egoX)	For students with higher achievement to create and maintain more ties
Self-concept alter (altX)	To create and maintain more ties to students with higher achievement
Self-concept homophily (simX)	For actors to be tied to those with similar achievement
Dependent variable: general academic self-concept (ego maintains, increases or decreases his or her self-concept)	
Linear shape (linear)	For self-concept to increase or decrease over time
Quadratic shape (quad)	The effect of self-concept on itself (the higher initial self-concept, the higher increase)
Gender (effFrom)	For girls to increase/decrease their self-concept (compared to boys)
SES (effFrom)	For actors with a higher SES to increase/decrease their self-concepts
Ethnic background	
Turkish (effFrom)	For actors with Turkish minority background to increase/decrease their self-concept (compared to those with a majority background)

Easter-European (effFrom)	For actors with Eastern-European minority background to increase/decrease their self-concept (compared to those with a majority background)
Other minority (effFrom)	For actors with another minority background to increase/decrease their self-concept (compared to those with a majority background)
Individual achievement (effFrom)	For actors with higher achievement to increase/decrease their self-concepts
Av. achievement of whole classroom (BFLPE) (effFrom)	For actors in classes with higher average achievement to increase/decrease their self-concepts
Av. achievement of nominated peers	
friends (avXAlt)	For actors with higher-achieving friends to increase/decrease their self-concepts
study partners (avXAlt)	For actors with higher-achieving study partners to increase/decrease their self-concepts
popular students (avXAlt)	For actors who perceive higher achieving students as popular to increase/decrease their self-concepts
Av. achievement of same-gender classmates (effFrom)	For actors in classes where same-gender classmates have higher average achievement to increase/decrease their self-concepts
Av. achievement of same-ethnic classmates (effFrom)	For actors in classes where same-ethnic classmates have higher average achievement to increase/decrease their self-concepts

Note. For the sake of simplicity, the descriptions focus only on friendship nominations but the effects work similarly for the nomination of co-study peers and the nomination of students as popular.

Supplement 3: Full model results

Table S4

SAOMs predicting changes in friendship status (network dynamics) and academic self-concept (behavior dynamics): full results (same models as reported in Table 1 of the manuscript)

	Model F.1		Model F.2		Model F.3		Model F.4		Model F.5	
	Est.	CI	Est.	CI	Est.	CI	Est.	CI	Est.	CI
**Network dynamics: Tie selection**										
Outdegree (density)	**-1.08**	[-1.24 - -0.93]	**-1.06**	[-1.23 - -0.9]	**-1.11**	[-1.22 - -0.95]	**-1.08**	[-1.23 - -0.94]	**-1.07**	[-1.21 - -0.93]
Reciprocity	**1.74**	[1.65 - 1.83]	**1.73**	[1.65 - 1.82]	**1.74**	[1.63 - 1.82]	**1.73**	[1.64 - 1.83]	**1.74**	[1.66 - 1.83]
Transitive triplets	**0.55**	[0.53 - 0.58]	**0.55**	[0.52 - 0.58]	**0.55**	[0.53 - 0.58]	**0.55**	[0.52 - 0.58]	**0.55**	[0.52 - 0.58]
Transitive reciprocal triplets	**-0.27**	[-0.31 - -0.23]	**-0.27**	[-0.3 - -0.23]	**-0.27**	[-0.31 - -0.23]	**-0.27**	[-0.31 - -0.23]	**-0.27**	[-0.3 - -0.23]
Indegree: popularity	**0.02**	[0 - 0.03]	**0.02**	[0 - 0.03]	**0.02**	[0.01 - 0.03]	**0.02**	[0 - 0.03]	**0.02**	[0 - 0.03]
Outdegree: activity	**-0.22**	[-0.24 - -0.2]	**-0.22**	[-0.24 - -0.2]	**-0.22**	[-0.23 - -0.2]	**-0.22**	[-0.24 - -0.2]	**-0.22**	[-0.24 - -0.2]
Gender (female): ego	**-0.11**	[-0.18 - -0.04]	**-0.11**	[-0.19 - -0.05]	**-0.11**	[-0.17 - -0.05]	**-0.11**	[-0.18 - -0.04]	**-0.10**	[-0.18 - -0.02]
Gender (female): alter	**0.06**	[0 - 0.11]	**0.06**	[0 - 0.11]	**0.06**	[-0.01 - 0.12]	**0.06**	[-0.01 - 0.12]	**0.05**	[-0.01 - 0.12]
Gender homophily (same gender)	**0.30**	[0.25 - 0.36]	**0.30**	[0.25 - 0.36]	**0.31**	[0.24 - 0.36]	**0.31**	[0.25 - 0.36]	**0.30**	[0.23 - 0.36]
SES homophily (similarity)	0.00	[-0.11 - 0.13]	0.00	[-0.12 - 0.12]	0.02	[-0.11 - 0.12]	0.00	[-0.13 - 0.12]	0.00	[-0.12 - 0.11]
Ethnic homophily (same ethnic background)	**0.12**	[0.08 - 0.17]	**0.12**	[0.07 - 0.16]	**0.12**	[0.08 - 0.17]	**0.12**	[0.07 - 0.18]	**0.12**	[0.07 - 0.17]
Achievement ego	0.03	[-0.01 - 0.07]	**0.03**	[0 - 0.07]	0.03	[-0.01 - 0.07]	**0.03**	[-0.01 - 0.07]	0.03	[-0.01 - 0.07]
Achievement alter	**0.06**	[0.03 - 0.1]	**0.07**	[0.03 - 0.1]	**0.06**	[0.03 - 0.1]	**0.06**	[0.03 - 0.1]	**0.06**	[0.03 - 0.1]
Achievement similarity	**0.46**	[0.24 - 0.7]	**0.45**	[0.24 - 0.66]	**0.45**	[0.23 - 0.69]	**0.46**	[0.27 - 0.67]	**0.45**	[0.21 - 0.66]
ASC ego	0.02	[-0.01 - 0.05]	0.02	[-0.01 - 0.05]	0.01	[-0.01 - 0.04]	0.02	[-0.01 - 0.04]	0.02	[-0.01 - 0.04]
ASC alter	0.00	[-0.03 - 0.02]	0.00	[-0.03 - 0.02]	0.00	[-0.02 - 0.02]	0.00	[-0.03 - 0.03]	0.00	[-0.02 - 0.02]
ASC similarity	**0.15**	[0.02 - 0.27]	**0.16**	[0.03 - 0.3]	**0.14**	[0.01 - 0.29]	**0.15**	[0.01 - 0.29]	**0.16**	[0.04 - 0.29]
**Behavior dynamics: Academic self-concept**										

linear shape	-0.01	[-0.06 - 0.04]	-0.01	[-0.06 - 0.03]	-0.02	[-0.06 - 0.03]	-0.02	[-0.06 - 0.03]	-0.02	[-0.06 - 0.03]
quadratic shape	0.00	[-0.03 - 0.03]	0.01	[-0.02 - 0.03]	0.00	[-0.02 - 0.03]	0.00	[-0.03 - 0.03]	0.00	[-0.02 - 0.03]
Gender (female)	-0.04	[-0.1 - 0.02]	**-0.06**	[-0.12 - 0]	-0.04	[-0.11 - 0.03]	-0.05	[-0.12 - 0.02]	-0.03	[-0.08 - 0.03]
SES	0.00	[0 - 0]	0.00	[0 - 0]	0.00	[0 - 0]	0.00	[0 - 0]	0.00	[0 - 0]
Ethnic origin: German (reference group)										
Ethnic origin: Turkish	**0.13**	[0.02 - 0.24]	**0.15**	[0.04 - 0.26]	**0.14**	[0.05 - 0.26]	**0.15**	[0.05 - 0.26]	**0.13**	[0.02 - 0.25]
Ethnic origin: Polish	0.00	[-0.11 - 0.11]	0.00	[-0.12 - 0.12]	-0.01	[-0.12 - 0.13]	0.00	[-0.11 - 0.1]	-0.02	[-0.12 - 0.08]
Ethnic origin: Other	**0.08**	[0 - 0.16]	**0.09**	[0.01 - 0.17]	**0.08**	[0.01 - 0.16]	**0.09**	[0.02 - 0.17]	0.07	[-0.01 - 0.15]
Individual Achievement	**0.31**	[0.26 - 0.38]	**0.26**	[0.21 - 0.31]	**0.28**	[0.24 - 0.32]	**0.28**	[0.23 - 0.34]	**0.28**	[0.24 - 0.33]
Av. Achievement of whole classroom (BFLPE)	**-0.23**	[-0.31 - -0.15]			**-0.21**	[-0.37 - -0.11]	**-0.17**	[-0.39 - 0.03]	**-0.16**	[-0.29 - -0.04]
Av. achievement of nominated peers										
friends			**-0.10**	[-0.16 - -0.03]	0.03	[-0.07 - 0.14]	0.06	[-0.05 - 0.17]	0.05	[-0.05 - 0.14]
Av. achievement of same-gender classmates							-0.06	[-0.24 - 0.12]		
Av. achievement of same-ethnic classmates									-0.07	[-0.19 - 0.04]

Note. Bold print = significant (p < .05)

Table S5

SAOMs predicting the selection of study partners (S) and the perception of popular peers (P) as well as academic self-concepts (behavior dynamics): full results (same models as reported in Table 2 of the manuscript)

	Model S.2		Model S.3		Model P.2		Model P.3	
	Est.	CI	Est.	CI	Est.	CI	Est.	CI
**Network dynamics: Tie selection**								
Outdegree (density)	**-3.11**	[-3.27 - -2.95]	**-3.10**	[-3.29 - -2.91]	**-2.50**	[-2.63 - -2.38]	**-2.49**	[-2.6 - -2.39]
Reciprocity	**1.90**	[1.78 - 2]	**1.90**	[1.8 - 2.03]	**0.36**	[0.27 - 0.46]	**0.36**	[0.27 - 0.44]
Transitive triplets	**0.84**	[0.76 - 0.91]	**0.84**	[0.77 - 0.91]	**0.27**	[0.24 - 0.31]	**0.27**	[0.24 - 0.31]
Transitive reciprocal triplets	**-0.84**	[-0.96 - -0.72]	**-0.85**	[-0.96 - -0.75]	-0.04	[-0.09 - 0.02]	-0.04	[-0.09 - 0.02]
Indegree: popularity	**-0.05**	[-0.08 - -0.02]	**-0.05**	[-0.09 - -0.01]	**0.13**	[0.12 - 0.14]	**0.13**	[0.12 - 0.14]
Outdegree: activity	**0.08**	[0.07 - 0.09]	**0.08**	[0.07 - 0.09]	0.01	[-0.01 - 0.02]	0.01	[-0.01 - 0.02]
Gender: ego	0.03	[-0.07 - 0.12]	0.03	[-0.05 - 0.11]	-0.03	[-0.08 - 0.02]	-0.03	[-0.08 - 0.01]
Gender: alter	0.05	[-0.04 - 0.13]	0.05	[-0.05 - 0.14]	**-0.07**	[-0.12 - -0.03]	**-0.08**	[-0.13 - -0.03]
Gender homophily (same gender)	**0.68**	[0.62 - 0.75]	**0.68**	[0.59 - 0.75]	**0.34**	[0.29 - 0.39]	**0.34**	[0.29 - 0.39]
SES homophily (similarity)	**0.14**	[-0.03 - 0.3]	0.14	[-0.03 - 0.33]	0.10	[-0.03 - 0.22]	0.08	[-0.04 - 0.22]
Ethnic homophily (same ethnic background)	**0.19**	[0.12 - 0.26]	**0.20**	[0.14 - 0.26]	**0.06**	[0.01 - 0.11]	**0.06**	[0.01 - 0.11]
Achievement ego	**-0.06**	[-0.1 - -0.02]	**-0.07**	[-0.11 - -0.02]	**-0.04**	[-0.07 - -0.01]	**-0.04**	[-0.07 - -0.01]
Achievement alter	**0.08**	[0.03 - 0.12]	**0.06**	[0.02 - 0.11]	0.01	[-0.02 - 0.04]	0.01	[-0.02 - 0.04]
Achievement similarity	**0.46**	[0.11 - 0.78]	**0.47**	[0.12 - 0.74]	0.17	[-0.08 - 0.42]	0.16	[-0.06 - 0.38]
ASC ego	**0.08**	[0.05 - 0.12]	**0.08**	[0.05 - 0.12]	0.02	[-0.01 - 0.05]	0.02	[-0.01 - 0.04]
ASC alter	0.01	[-0.02 - 0.05]	0.02	[-0.02 - 0.05]	**0.02**	[0 - 0.05]	**0.02**	[0 - 0.04]
ASC similarity	0.16	[-0.04 - 0.33]	0.11	[-0.08 - 0.29]	-0.01	[-0.14 - 0.12]	-0.02	[-0.15 - 0.12]
**Behavior dynamics: Academic self-concept**								
linear shape	-0.01	[-0.05 - 0.04]	-0.02	[-0.06 - 0.03]	-0.01	[-0.06 - 0.03]	-0.01	[-0.06 - 0.03]
quadratic shape	0.01	[-0.02 - 0.03]	0.00	[-0.03 - 0.03]	0.00	[-0.02 - 0.03]	0.00	[-0.02 - 0.03]

Gender (female)	-0.05	[-0.12 - 0.02]	-0.02	[-0.07 - 0.04]	-0.05	[-0.11 - 0.01]	-0.03	[-0.1 - 0.03]
SES	**0.00**	[0 - 0]	0.00	[0 - 0]	0.00	[0 - 0]	0.00	[0 - 0]
Ethnic origin: German (reference group)								
Ethnic origin: Turkish	**0.15**	[0.05 - 0.24]	**0.16**	[0.07 - 0.27]	**0.15**	[0.05 - 0.25]	**0.13**	[0.04 - 0.22]
Ethnic origin: Polish	0.00	[-0.13 - 0.13]	-0.01	[-0.1 - 0.1]	0.01	[-0.1 - 0.11]	-0.01	[-0.11 - 0.12]
Ethnic origin: Other	**0.10**	[0.01 - 0.18]	**0.09**	[0 - 0.17]	**0.09**	[0.01 - 0.17]	**0.08**	[0.01 - 0.16]
Individual Achievement	**0.25**	[0.21 - 0.29]	**0.28**	[0.24 - 0.33]	**0.27**	[0.22 - 0.32]	**0.28**	[0.24 - 0.32]
Av. Achievement of whole classroom (BFLPE)			**-0.25**	[-0.34 - -0.15]			**-0.16**	[-0.27 - -0.01]
Av. achievement of nominated peers								
homework peers	-0.05	[-0.12 - 0.03]	**0.11**	[0.02 - 0.2]				
popular peers					**-0.16**	[-0.24 - -0.09]	-0.02	[-0.17 - 0.09]

Note. Fat = significant (p < .05)

Supplement 4: Robustness checks for operationalization of achievement

Table S6

SAOMs predicting changes in friendship status (network dynamics) and academic self-concept (behavior dynamics): full results for Model F.3 (see Supplement C and manuscript) using only grades and only test-scores as achievement indicators

	**Achievement based on grades (lower = higher ach.)**		**Achievement based on tests**	
	Est.	CI	Est.	CI
**Network dynamics: Tie selection**				
Outdegree (density)	**-1.04**	[-1.18 - -0.9]	**-1.08**	[-1.25 - -0.91]
Reciprocity	**1.74**	[1.65 - 1.84]	**1.75**	[1.65 - 1.84]
Transitive triplets	**0.56**	[0.52 - 0.59]	**0.55**	[0.52 - 0.58]
Transitive reciprocal triplets	**-0.27**	[-0.32 - -0.23]	**-0.27**	[-0.31 - -0.23]
Indegree: popularity	**0.02**	[0 - 0.03]	**0.02**	[0 - 0.04]
Outdegree: activity	**-0.22**	[-0.24 - -0.2]	**-0.22**	[-0.24 - -0.2]
Gender (female): ego	**-0.12**	[-0.19 - -0.06]	**-0.12**	[-0.19 - -0.06]
Gender (female): alter	0.04	[-0.02 - 0.09]	**0.06**	[0.01 - 0.13]
Gender homophily (same gender)	**0.30**	[0.24 - 0.36]	**0.30**	[0.24 - 0.36]
Ethnic homophily (same ethnic background)	**0.12**	[0.08 - 0.17]	**0.12**	[0.07 - 0.17]
SES homophily (similarity)	0.01	[-0.12 - 0.12]	0.01	[-0.1 - 0.12]
Achievement ego	**-0.03**	[-0.07 - 0]	**0.03**	[0 - 0.07]
Achievement alter	**-0.07**	[-0.1 - -0.04]	**0.06**	[0.03 - 0.1]
Achievement similarity	**0.33**	[0.11 - 0.55]	**0.45**	[0.23 - 0.66]
ASC ego	0.02	[-0.01 - 0.04]	0.02	[-0.01 - 0.05]
ASC alter	-0.01	[-0.04 - 0.01]	0.00	[-0.03 - 0.02]
ASC similarity	**0.11**	[-0.02 - 0.25]	**0.12**	[-0.02 - 0.25]
**Behavior dynamics: Academic self-concept**				
linear shape	-0.02	[-0.06 - 0.03]	-0.01	[-0.06 - 0.04]
quadratic shape	-0.02	[-0.05 - 0.02]	0.01	[-0.02 - 0.03]
Gender (female)	**-0.17**	[-0.24 - -0.1]	-0.06	[-0.13 - 0.01]
SES	0.00	[0 - 0]	0.00	[0 - 0]
Ethnic origin: German (reference group)				
Ethnic origin: Turkish	0.07	[-0.02 - 0.17]	**0.13**	[0.02 - 0.24]
Ethnic origin: Polish	-0.03	[-0.14 - 0.09]	-0.02	[-0.12 - 0.09]
Ethnic origin: Other	0.01	[-0.07 - 0.09]	0.05	[-0.03 - 0.14]
Individual Achievement	**-0.30**	[-0.34 - -0.27]	**0.25**	[0.21 - 0.3]
Av. Achievement of whole classroom (BFLPE)	**0.17**	[0.04 - 0.31]	**-0.17**	[-0.29 - -0.04]
Av. achievement of nominated peers	-0.02	[-0.12 - 0.07]	0.01	[-0.08 - 0.12]

Note. Bold print = significant ($p < .05$)

Table S7

SAOMs predicting changes in study partner status (network dynamics) and academic self-concept (behavior dynamics): full results for Model S.3 (see Supplement C and manuscript) using only grades and only test-scores as achievement indicators

	**Achievement based on grades (lower = higher ach.)**		**Achievement based on tests**	
	Est.	CI	Est.	CI
**Network dynamics: Tie selection**				
Outdegree (density)	**-3.03**	[-3.18 - -2.89]	**-3.03**	[-3.19 - -2.84]
Reciprocity	**1.88**	[1.77 - 2]	**1.91**	[1.8 - 2.02]
Transitive triplets	**0.81**	[0.75 - 0.88]	**0.85**	[0.78 - 0.92]
Transitive reciprocal triplets	**-0.80**	[-0.91 - -0.69]	**-0.86**	[-0.97 - -0.75]
Indegree: popularity	**-0.07**	[-0.11 - -0.03]	**-0.06**	[-0.11 - -0.02]
Outdegree: activity	**0.08**	[0.07 - 0.08]	**0.07**	[0.06 - 0.08]
Gender (female): ego	0.02	[-0.06 - 0.12]	0.02	[-0.06 - 0.09]
Gender (female): alter	0.01	[-0.07 - 0.08]	0.06	[-0.03 - 0.15]
Gender homophily (same gender)	**0.67**	[0.59 - 0.77]	**0.67**	[0.6 - 0.75]
Ethnic homophily (same ethnic background)	**0.20**	[0.12 - 0.27]	**0.18**	[0.12 - 0.25]
SES homophily (similarity)	0.14	[-0.04 - 0.34]	0.12	[-0.03 - 0.29]
Achievement ego	0.00	[0 - 0]	**-0.07**	[-0.11 - -0.03]
Achievement alter	**-0.12**	[-0.16 - -0.09]	**0.07**	[0.03 - 0.12]
Achievement similarity	**0.60**	[0.31 - 0.89]	**0.46**	[0.14 - 0.76]
ASC ego	**0.07**	[0.04 - 0.11]	**0.09**	[0.05 - 0.13]
ASC alter	-0.01	[-0.05 - 0.03]	0.01	[-0.02 - 0.05]
ASC similarity	0.08	[-0.1 - 0.25]	0.08	[-0.08 - 0.26]
**Behavior dynamics: Academic self-concept**				
linear shape	-0.03	[-0.08 - 0.02]	-0.01	[-0.06 - 0.03]
quadratic shape	-0.02	[-0.05 - 0.01]	0.00	[-0.03 - 0.03]
Gender (female)	**-0.19**	[-0.26 - -0.12]	-0.06	[-0.12 - 0.01]
SES	0.00	[0 - 0]	0.00	[0 - 0]
Ethnic origin: German (reference group)				
Ethnic origin: Turkish	0.07	[-0.03 - 0.17]	**0.12**	[0.01 - 0.23]
Ethnic origin: Polish	-0.03	[-0.17 - 0.07]	-0.03	[-0.15 - 0.08]
Ethnic origin: Other	0.01	[-0.07 - 0.08]	0.05	[-0.04 - 0.13]
Individual Achievement	**-0.30**	[-0.35 - -0.26]	**0.26**	[0.2 - 0.3]
Av. Achievement of whole classroom (BFLPE)	**0.20**	[0.08 - 0.3]	**-0.21**	[-0.31 - -0.11]
Av. achievement of nominated peers	-0.07	[-0.16 - 0.03]	0.07	[-0.04 - 0.2]

Note. Bold print = significant (p < .05)

Table S8

SAOMs predicting changes in popularity perception (network dynamics) and academic self-concept (behavior dynamics): full results for Model P.3 (see Supplement C and manuscript) using only grades and only test-scores as achievement indicators

	**Achievement based on grades (lower = higher ach.)**		**Achievement based on tests**	
	Est.	CI	Est.	CI
**Network dynamics: Tie selection**				
Outdegree (density)	**-2.46**	[-2.57 - -2.34]	**-2.42**	[-2.52 - -2.31]
Reciprocity	**0.37**	[0.28 - 0.46]	**0.36**	[0.26 - 0.44]
Transitive triplets	**0.27**	[0.24 - 0.31]	**0.28**	[0.25 - 0.3]
Transitive reciprocal triplets	-0.04	[-0.09 - 0.02]	-0.03	[-0.08 - 0.03]
Indegree: popularity	**0.13**	[0.12 - 0.14]	**0.13**	[0.12 - 0.13]
Outdegree: activity	0.00	[-0.01 - 0.02]	0.00	[-0.02 - 0.01]
Gender (female): ego	-0.02	[-0.07 - 0.03]	-0.03	[-0.08 - 0.02]
Gender (female): alter	**-0.08**	[-0.13 - -0.03]	**-0.08**	[-0.13 - -0.03]
Gender homophily (same gender)	**0.34**	[0.29 - 0.39]	**0.33**	[0.28 - 0.39]
Ethnic homophily (same ethnic background)	**0.06**	[0.01 - 0.11]	**0.07**	[0.02 - 0.12]
SES homophily (similarity)	0.08	[-0.04 - 0.19]	0.09	[-0.02 - 0.2]
Achievement ego	0.01	[-0.02 - 0.04]	**-0.04**	[-0.07 - -0.01]
Achievement alter	-0.02	[-0.05 - 0.01]	0.01	[-0.02 - 0.04]
Achievement similarity	**0.21**	[0 - 0.44]	0.14	[-0.07 - 0.35]
ASC ego	0.02	[-0.01 - 0.04]	0.02	[-0.01 - 0.04]
ASC alter	**0.02**	[0 - 0.05]	**0.03**	[0.01 - 0.05]
ASC similarity	0.02	[-0.1 - 0.15]	0.01	[-0.12 - 0.13]
**Behavior dynamics: Academic self-concept**				
linear shape	-0.01	[-0.06 - 0.04]	-0.01	[-0.06 - 0.04]
quadratic shape	-0.02	[-0.04 - 0.01]	0.01	[-0.03 - 0.03]
Gender (female)	**-0.17**	[-0.24 - -0.1]	**-0.07**	[-0.13 - -0.01]
SES	0.00	[0 - 0]	0.00	[0 - 0]
Ethnic origin: German (reference group)				
Ethnic origin: Turkish	0.07	[-0.03 - 0.16]	**0.13**	[0.04 - 0.24]
Ethnic origin: Polish	-0.02	[-0.13 - 0.1]	-0.02	[-0.13 - 0.1]
Ethnic origin: Other	0.02	[-0.07 - 0.11]	0.04	[-0.04 - 0.13]
Individual Achievement	**-0.31**	[-0.35 - -0.26]	**0.26**	[0.21 - 0.3]
Av. Achievement of whole classroom (BFLPE)	0.08	[-0.06 - 0.23]	-0.10	[-0.24 - 0.04]
Av. achievement of nominated peers	0.10	[-0.03 - 0.21]	-0.06	[-0.19 - 0.07]

Note. Bold print = significant (p < .05)

Supplement 5: Robustness check for operationalization of self-concept

Table S9

SAOMs predicting changes in friendship status (network dynamics) and academic self-concept (behavior dynamics): full results for Model F.3 (see Supplement C and manuscript) using a latent self-concept factor with correlated residuals between the two self-efficacy items and between the two items relating to language domains

	**Self-Concept variable with residual correlations**	
	Est.	CI
**Network dynamics: Tie selection**		
Outdegree (density)	**-1.09**	[-1.27 - -0.92]
Reciprocity	**1.74**	[1.64 - 1.83]
Transitive triplets	**0.55**	[0.53 - 0.58]
Transitive reciprocal triplets	**-0.27**	[-0.31 - -0.24]
Indegree: popularity	**0.02**	[0.01 - 0.03]
Outdegree: activity	**-0.22**	[-0.24 - -0.2]
Gender (female): ego	**-0.11**	[-0.18 - -0.05]
Gender (female): alter	**0.06**	[0 - 0.12]
Gender homophily (same gender)	**0.30**	[0.24 - 0.36]
Ethnic homophily (same ethnic background)	**0.13**	[0.08 - 0.17]
SES homophily (similarity)	-0.01	[-0.14 - 0.12]
Achievement ego	**0.03**	[0 - 0.07]
Achievement alter	**0.07**	[0.03 - 0.09]
Achievement similarity	**0.45**	[0.23 - 0.71]
ASC ego	0.02	[-0.01 - 0.04]
ASC alter	0.00	[-0.02 - 0.02]
ASC similarity	**0.13**	[0 - 0.26]
linear shape	-0.01	[-0.06 - 0.04]
quadratic shape	0.01	[-0.02 - 0.03]
Gender (female)	**-0.07**	[-0.13 - -0.01]
SES	0.00	[0 - 0]
Ethnic origin: German (reference group)		
Ethnic origin: Turkish	**0.13**	[0.02 - 0.25]
Ethnic origin: Polish	-0.01	[-0.12 - 0.09]
Ethnic origin: Other	0.06	[-0.03 - 0.15]
Individual Achievement	**0.25**	[0.2 - 0.29]
Av. Achievement of whole classroom (BFLPE)	**-0.16**	[-0.28 - -0.05]
Av. achievement of nominated peers	0.01	[-0.08 - 0.11]

Note. Bold print = significant (p < .05)
